# Fecal Microbiota Transplantation (FMT) Alleviates Experimental Colitis in Mice by Gut Microbiota Regulation

**DOI:** 10.4014/jmb.2002.02044

**Published:** 2020-05-13

**Authors:** Wanying Zhang, Guiling Zou, Bin Li, Xuefei Du, Zhe Sun, Yu Sun, Xiaofeng Jiang

**Affiliations:** 1 Department of Clinical Laboratory, Fourth Affiliated Hospital of Harbin Medical University, 37 Yiyuan Street, Nangang District, Harbin 150001, P.R. China; 2 Heilongjiang Longwei Precision Medical Laboratory Center, Longchuan Road, Songbei District, Harbin 150028, P.R. China

**Keywords:** Ulcerative colitis (UC), fcal microbiota transplantation (FMT), gut microbiota, NF-κB signaling pathway

## Abstract

Inflammatory bowel disease (IBD) is an increasing global burden and a predisposing factor to colorectal cancer. Although a number of treatment options are available, the side effects could be considerable. Studies on fecal microbiota transplantation (FMT) as an IBD intervention protocol require further validation as the underlying mechanisms for its attenuating effects remain unclear. This study aims to demonstrate the ameliorative role of FMT in an ulcerative colitis (UC) model induced by dextran sulfate sodium (DSS) and elucidate its relative mechanisms in a mouse model. It was shown that FMT intervention decreased disease activity index (DAI) levels and increased the body weight, colon weight and colon length of experimental animals. It also alleviated histopathological changes, reduced key cytokine expression and oxidative status in the colon. A down-regulated expression level of genes associated with NF-κB signaling pathway was also observed. The results of 16S rRNA gene sequencing showed that FMT intervention restored the gut microbiota to the pattern of the control group by increasing the relative abundance of Firmicutes and decreasing the abundances of Bacteroidetes and Proteobacteria. The relative abundances of the genera *Lactobacillus*, *Butyricicoccus*, *Lachnoclostridium*, *Olsenella* and *Odoribacter* were upregulated but *Helicobacter*, *Bacteroides* and *Clostridium* were reduced after FMT administration. Furthermore, FMT administration elevated the concentrations of SCFAs in the colon. In conclusion, FMT intervention could be suitable for UC control, but further validations via clinical trials are recommended.

## Introduction

Inflammatory bowel disease (IBD) is a severe immune-mediated condition that has been implicated in the manifestation of colorectal cancer [1]. Characterized by chronic episodes of remissions and relapses, IBD is a growing 21^st^ century disease, especially in newly industrialized countries cutting across several continents whose societies are becoming westernized [2, 3]. A major predisposing factor is an imbalance in the host’s mucosal immune system response to bacterial antigens which results in repeated secretion of pro-inflammatory cytokines [4]. Given the key roles that the gut microbiota plays in pathogen suppression and overall health, it is thus unsurprising that research interventions aimed at promoting this healthier microbial ecosystem have been on the increase, with the potential to alleviate ulcerative colitis (UC) and Crohn’s disease (CD) among sufferers [5].

Although inflammation is the immune system’s response to a number of deleterious activities such as pathogen- infected cells, the pathways involved can exacerbate pathogenic processes and result in increased host susceptibility to attack [6]. Some cytokines like TNF-α, IL-6 and IL-1β have been reported to be genes commonly targeted by NF-κB [7]. Enshrined in the cytoplasm by inhibitor proteins (IκBs), NF-κB is translocated to the nucleus where downstream target genes are initiated for pro-inflammatory signaling, which occur by a series of complex phosphorylation and degradation processes [8]. The NF-κB protein complex pathway has been implicated in UC and CD onset in humans as well as in laboratory trials [9, 10].

A complex interaction exists between the gut microbiota and UC pro-inflammatory factors [11]. It has been demonstrated that UC pathogenesis can be mitigated by a balanced interplay of beneficial microbes and host genetic factors as well as prevailing environmental triggers [12]. The fecal microbiota transplantation (FMT) protocol is new but has been shown to improve structural modulation of the gut microbiota, thus alleviating IBD and chronic gastrointestinal dysbiosis [13, 14]. However, the specific mechanisms by which the FMT protocol alleviates UC are still unclear, and most of what has been reported so far are partial case reports and studies. Although a recent study using a UC mouse model induced with dextran sulfate sodium (DSS) showed that the intestinal flora was similar to that of IBD in humans, more in vivo studies are needed to further validate previous claims [15].

The present study aims to evaluate the alleviative effects of FMT on inflammatory responses in a DSS-induced colitis model by highlighting the mechanisms involved. Therein, it was shown that FMT effectively reduced the disease activity index (DAI), improved key cytokine expression and oxidative status, reversed the histopathology and symptoms of UC and altered the levels of representative bacteria and their metabolites. These results will provide further insight into the specific mechanistic roles that FMT plays in alleviating UC and may provide a sound scientific basis for future related studies.

## Materials and Methods

### Animals and Experimental Design

A total of 36 female BALB/c mice (7 weeks old, 18-20 g weight) were purchased from the Vital River Laboratory Animal Technology Company (China) and raised at the Harbin Medical University Animal Center. Male mice were not used as their potential aggressiveness could constitute an uncontrolled variable in group-housed male mice. Mice were allowed one week to acclimatize prior to the study. For this period, food and water were given ad libitum and the room was ventilated, having an ambient temperature of 22 °C ± 1°C with 50% ± 10% humidity and a 12-h diurnal light cycle (lights on 07:00–19:00). All animal experiments reported in this study were approved by the Animal Care and Ethics Committee of Harbin Medical University (2019-SCILLSC-06).

**Fig. 1:**
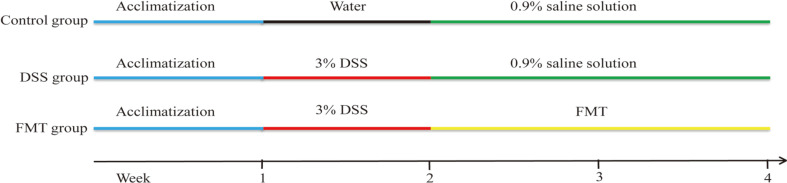
The animal experimental protocol.

The animal experimental protocol was shown in Fig. 1. Mice were randomly divided into 3 groups, namely the i) control group, ii) DSS group, and iii) FMT group. Firstly, 3% DSS solution as their freely provided drinking water was administered to all mice for 7 days except for the control group to induce the UC model. Fecal suspension was prepared as previously described [16]. Briefly, fresh fecal pellets (200 mg) from mice in the control group were collected every day, and resuspended in a 5 ml saline solution, vigorously shaken for 3 min and allowed to settle by gravity for 2 min. FMT administration was performed by gavage with 200 μl of the supernatant from the fecal sample once a day for a period of 2 weeks in the FMT group. At the same time, the mice in the control and DSS groups were gavaged by 200 μl 0.9% saline solution.

On the last day of study, the mice were sacrificed under humane conditions, the colons were opened up, and fecal contents were then removed by gentle pressure with a pair of forceps. The distal portion (approximately 30 mm) of the colons and other sections were selected for the hematoxylin-eosin (HE) staining and the western- blotting examination respectively. Colonic samples from the different groups were collected for pro- inflammatory cytokine examination.

### Disease Activity Index (DAI)

In the intervention period, the body weight and stool consistency of mice were observed on time. DAI was scored as earlier described (Table S1) [17, 18]. After intervention, animals were humanely sacrificed by cervical dislocation, and the colons were removed. Colon lengths and weights were measured using a ruler and electronic analytical balance, respectively.

### Histopathological Symptoms

To observe detailed histopathological changes, the colons of different mice were first stored in a 10%-buffered formalin solution. These were then embedded in paraffin, cut into 5 μm sections, stained with hematoxylin-eosin, and then placed under a light microscope for examination.

### Cytokine Analysis

Total RNA was extracted from colonic tissues using the RNAiso Plus Kit (Takara, China). RNA purification (DSS removal) was conducted using lithium chloride as previously described, because it inhibits real-time quantitative PCR (RT-qPCR) amplification [19]. Afterwards, reverse-transcription of RNA into cDNA was performed using the PrimeScriptTM RT Reagent Kit with gDNA Eraser (Takara). Then, the quantification (qRT- PCR) of relative mRNA concentrations (TNF-α, IL-6, IL-1β and glyceraldehyde 3-phosphate dehydrogenase (GAPDH)) mRNA was performed using a 7500 Fast Real-Time PCR System (Applied Biosystems, USA) and a SYBR Green PCR Master Mix Kit (Takara) following the manufacturer’s protocol (Table S2). Expression level was normalized using GAPDH as a reference gene.

### Measurement of Colonic Oxidative Stress Parameters

Colonic tissue parameters like malondialdehyde (MDA) levels, total antioxidant capacity (T-AOC), catalase (CAT) and superoxide dismutase (SOD) activities were determined with standard assay kits (Nanjing Jiancheng Bioengineering Institute, China) following the manufacturer’s instructions.

### Microbial Analysis of Colonic Contents

Following the manufacturer’s instructions, a QIAamp DNA Stool Mini Kit (Qiagen, Germany) was used to extract the total microbiota genomic DNA from the colonic contents of the control, DSS and FMT groups, respectively. The V4 hypervariable region of the bacterial 16S rRNA gene was amplified using modified fusion primers 515F (5'-GTGCCAGCMGCCGCGGTAA-3') and 806R (5'-GGACTACHVGGGTWTCTAAT-3'), containing a 6-bp error-correcting barcode. In addition, the Illumina Miseq (Illumina, USA) protocol was used to prepare the PCR amplicons. Data obtained from these procedures were then merged with the FLASH 1.2.7 software [20]. The UCHIME algorithm was used to obtain the high-quality clean tags after discarding the chimera sequences [21]. Tags were clustered into distinct operational taxonomic units (OTUs) using Uparse software with a 97% sequence identity [22]. OTUs were classified using QIIME 1.7.0 against a curated database derived from GreenGenes [23]. The raw data were deposited as Sequence Read Archive (SRA) in National Coalition Building Institute (NCBI) under accession no. PRJNA624253.

### Short-Chain Fatty Acids (SCFAs) Analysis

The levels of SCFAs were measured as described previously by Li *et al.* [24] with some modifications. All the samples (50 mg) were pretreated by mixing deionized water (dH_2_O), homogenization (35 Hz, 4 min), ultrasound and centrifugation (5,000 g, 20 min, 4°C). Then 0.1 ml 50% H_2_SO_4_ and 0.5 ml of 2-methylvaleric acid (internal standard) were added to the supernatant obtained (0.8 ml), vortexed for 10 sec and oscillated in 5 min. The mixtures were centrifuged for 15 min at 12,000 g, 4°C, kept at −20°C for 30 min and transferred into a fresh 2 mL glass vial, for GC-MS analysis. GC-MS analysis was performed using an Agilent 7890B gas chromatograph system coupled with an Agilent 5977B mass spectrometer. The system utilized an HP-FFAP capillary column (J&W Scientific, USA).

### Western-Blotting Analysis

An ice-cold radio-immunoprecipitation assay (RIPA) buffer with 1% protease inhibitor and 1% phosphatase inhibitor (Beyotime Institute of Biotechnology, China) was used to homogenize colonic tissues obtained from the control, DSS and FMT groups. Homogenate was centrifuged at 12,000 g at 4°C for 15 min to obtain total protein and its concentrations were measured with the bicinchoninic acid protein assay (BCA, Beyotime, China) method. Sample electrophoresis was carried out on SDS-PAGE gel and then placed on 0.45 μm polyvinylidene fluoride membranes. Membranes were kept for 2 h with 5% fresh nonfat milk at room temperature before incubation with the primary antibodies at 4°C overnight as follows: IκBα (1:400, Wanleibio, China), p-IκBα (1:400, Wanleibio, China), p65 (1:500, Wanleibio), p-p65 (1:1,000, Wanleibio), and β-actin (1:500, Wanleibio,). After washing thrice with saline buffer and Tween, blots were incubated with horseradish peroxidase-conjugated secondary antibodies for 1 h at room temperature, and the immune complexes were detected by using an enhanced chemiluminescence kit (Beyotime Biotechnology). Lastly, the Gel-Pro Analyzer was used to quantify protein levels which appeared as luminescent bands.

### Statistical Analysis

Data from this study were analyzed using GraphPad Prism and SPSS software. A *p* value < 0.05 was considered statistically significant. In addition, all obtained data are expressed as the mean ± standard deviation (SD), with statistical significance analyzed using one-way ANOVA followed by a Duncan' test.

## Results and Discussion

### Effect of FMT on Growth Status and Clinical Symptoms

**Fig. 2:**
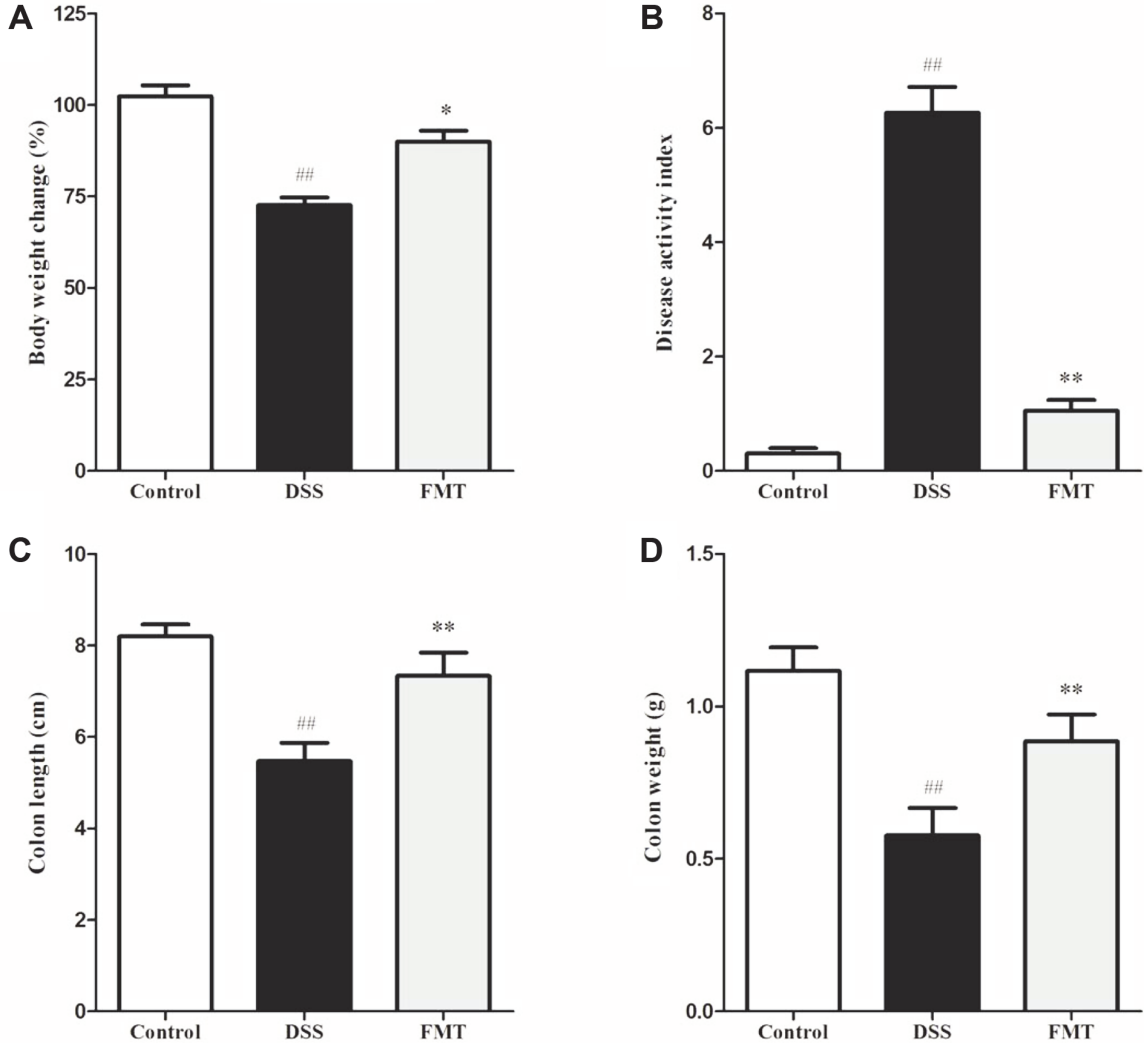
Effects of FMT on DSS-induced colitis mouse. (**A**) Changes in body weight (%) during the induction and recovery periods of colitis, (**B**) DAI values at the end of the recovery period, (**C**) Colon weight, and (**D**) Colon length. Data are presented as mean ± SD. ##*p* < 0.01 and #*p* < 0.05 vs the control group. **p < 0.01 and *p < 0.05 vs the DSS model group.

The DSS-induced colitis process causes ulceration of the intestinal epithelium of sufferers and among other complications, leads to disruption of the inner mucosal layer and an increased infiltration of acute, inflammatory immune cells [25, 26]. Some common acute UC parameters include body weight, DAI score, colon length and colon weight [27, 28]. The present study used these parameters to assess the impact of FMT regarding the onset and progression of colitis in the study animals. As shown in Fig. 2, the DSS group showed a significant decrease (*p* < 0.01) in body weight, colon weight and colon length compared with the control group. After FMT intervention, marked weight increase similar to the control group were observed (*p* < 0.05). In addition, FMT mice had significantly lower DAI score (1.05 ± 0.18) than that of the DSS group (6.26 ± 0.45). These findings were in line with the results of Burrello *et al.* [29], indicating that FMT intervention could effectively prevent DSS- induced UC.

### Effect of FMT on Colon Histopathological Alterations

**Fig. 3:**
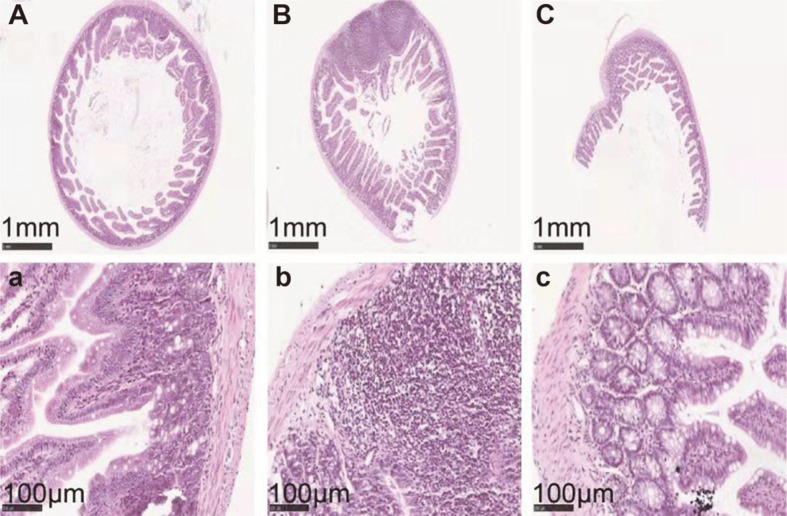
Effects of FMT intervention on the mouse colon histological changes (Representative hematoxylin and eosin staining). (**A**) Control group (× 50), (**B**) DSS model group (× 50), (**C**) FMT group (× 50), (**a**) Control group (× 400), (**b**) DSS model group (× 400), and (**c**) FMT group (× 400).

The histopathological status of observed colon samples are shown in Fig. 3. In the control group, normal colon histomorphology was visible (Figs. 3A and 3a). As anticipated, histolopathologic examination of samples from the DSS group showed ulceration, submucosal edema, cytoplasmic mucin depletion, mucosal infiltration, submucosal granulomatosis, and thickening of intestinal wall (Figs. 3B and 3b). After FMT intervention, symptoms of UC mice were pronouncedly relieved (Figs. 3C and 3c). Similar protective effects were also observed in an earlier study by Tian *et al.* [30].

### Effect of FMT on Oxidative Status in Colonic Tissue

Gut oxidative stress has been implicated in previous and recent UC studies. Phagocytes are triggered by pro- inflammatory factors which initiates reactive oxygen species (ROS) production [31]. Thus, protocols improving its antioxidant potentials may prevent UC onset and progression [32, 33]. In the DSS group of the present study, the levels of CAT, SOD and T-AOC were significantly decreased (*p* < 0.01), and the level of MDA increased significantly (*p* < 0.01) compared to the control group. SOD is required for breakdown of superoxide anion, which in turn catalyzes the reduction of O_2_^•−^ to H_2_O_2_, CAT then converts H_2_O_2_ to water [34]. Host T-AOC levels are important for optimal body functioning because they determine the degree of susceptibility to a range of diseases [35]. MDA level is a well-recognized biomarker of oxidative stress [36]. Based on these reports, the aforementioned results implied that oxidative stress activities occurred in the colon. After FMT intervention, the levels of CAT, SOD and T-AOC significantly increased (*p* < 0.05) and the level of MDA decreased (*p* < 0.01), implying that FMT intervention could effectively ameliorate oxidative stress.

### Effect of FMT on Pro-Inflammatory Cytokines and NF-κB Signaling Pathway in Colonic Tissue

**Fig. 5:**
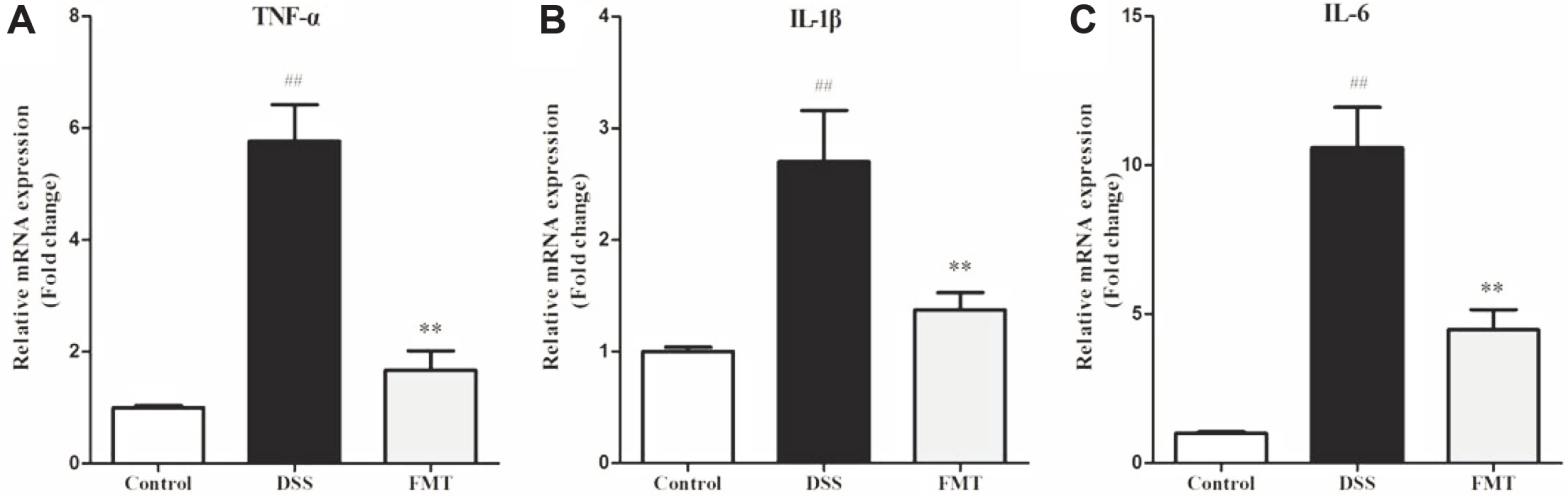
Effects of FMT administration on inflammatory gene expression of colonic tissues in DSS-induced colitis mouse. (**A**) TNF-α, (**B**) IL-1β, and (**C**) IL-6. Fold changes are presented as mean ± SD. ##*p* < 0.01 and #*p* < 0.05 vs the control group. **p < 0.01 and *p < 0.05 vs the DSS model group.

A well-known feature of the DSS-induced colitis is the excessive production of pro-inflammatory cytokines, resulting in severe degradation of the epithelium layer [37, 38]. Therefore, interventions that modulate the production of inflammatory cytokines can be promising therapeutic applications for colitis treatment. To evaluate the influence of FMT on cytokine expression in the UC mice induced by DSS, mRNA expression of pro- inflammatory cytokines was determined. As shown in Fig. 5, the mRNA levels of TNF-α, IL-1β and IL-6 were 5.76, 2.70 and 10.58 times higher in the DSS group than that in the control group, respectively. Pro-inflammatory cytokine levels (TNF-α, IL-6 and IL-1β) in colonic tissues have been previously reported to increase significantly by DSS exposure [39, 40]. In clinical applications, these markers are predictive of disease onset as they indicate variations in normal and pathogenic biological processes as well as evaluate the efficacy of attenuation protocols [5]. As a known pro-inflammatory cytokine, the tumor necrosis factor-α (TNF-α) induces apoptosis of epithelial cells, disrupts the epithelial barrier, and prolongs the inflammation reaction [41]. The expression of colonic IL-6 has been shown previously to increase after induced colitis with its antibody having beneficial effects [42]. Similarly, IL-1β is rapidly produced from innate immune cells in response to inflammation and infection [30]. These inflammatory cytokine mRNA levels were significantly inhibited by FMT intervention, which might suggest that FMT intervention had good anti-colitic effects by adjusting the levels of cytokines in colonic tissue and may be a therapeutic application for UC.

### Effect of FMT on the Composition of Colonic Microbiota

**Fig. 6:**
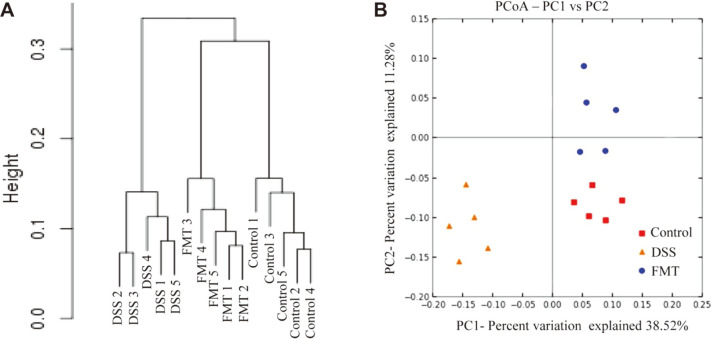
Influences of FMT administration on gut microbiota β-diversity. (**A**) Hierarchical clustering tree of weighted UniFrac distance, and (**B**) Principal coordinates analysis (PCoA) of Bray-Curtis distances.

The gut microbiota is a complex ecosystem with an array of bacteria genera which carry out a number of vital functions in the host, including maintaining intestinal homeostasis, upholding intestinal epithelial barrier, immune system development and providing necessary metabolic substrates for colonocytes. Imbalances in the gut microbial population have however been linked with many diseases, including UC and CD [43, 44]. The present study assessed the gut microbiota of the control, DSS and FMT groups using next-generation 16S rRNA gene sequencing protocol to determine whether or not gut microbiota changes were associated with the protective effects of FMT in DSS-induced mice. The hierarchical clustering analysis of weighted UniFrac distances revealed that the control and FMT groups were closely linked compared to the DSS group (Fig. 6A). Furthermore, the principal coordinate analysis (PCoA) results show that both FMT and control groups had similar gut microbiota structures which were clearly different from that of the DSS group (Fig. 6B).

**Fig. 7:**
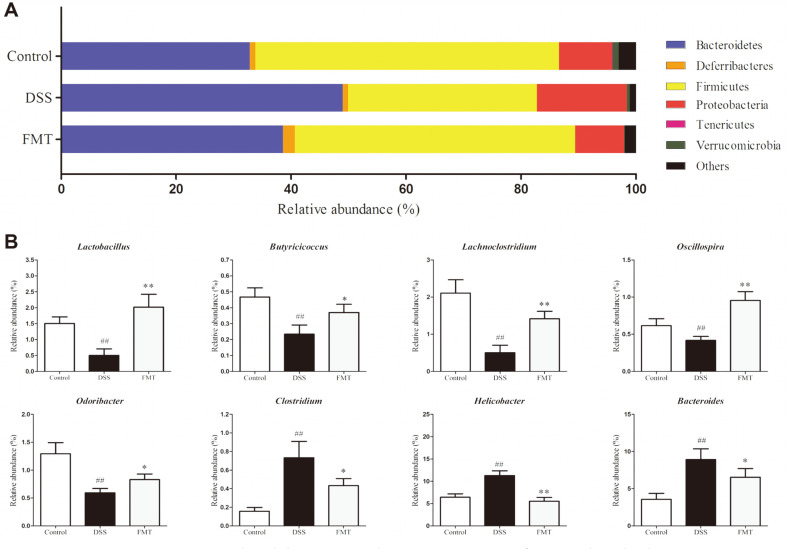
FMT intervention manipulated the gut microbiota composition of DSS-induced colitis mouse. (**A**) Gut microbiota distribution at the phylum level, and (**B**) Gut microbiota distribution at the genus level. Data are presented as mean ± SD. ##*p* < 0.01 and #*p* < 0.05 vs the control group. **p < 0.01 and *p < 0.05 vs the DSS model group.

In this study, the taxonomic composition of the microbiomes of the control, FMT and DSS groups was also carried out. The microbiota in samples analyzed from all studied groups were made up of Bacteriodetes, Firmicutes and Proteobacteria phyla. Compared to the control, we observed that DSS induction increased the relative abundances of Bacteriodetes (32.79-48.95%) and Proteobacteria (9.16-15.63%) respectively. In contrast, abundance of the Firmicutes phylum decreased from 52.80% to 32.85% (Fig. 7A). Similar changes of gut microbiota were reported previously [12, 45]. It has also been suggested that most microbes in the Firmicutes phylum have anti-inflammatory functions by modulating colon pH which inhibits pathogen growth. They also facilitate the production of SCFAs from non-digestible carbon sources known for pathogen suppression. An increase in the population of microbes in the Bacteroidetes phylum is known to produce significantly high lipopolysaccharide levels which in turn regulates pro-inflammatory mediators through the NF-κB and AKT pathways in colonic macrophages [46, 47]. After FMT treatment, the relative abundances of Bacteroidetes, Proteobacteria and Firmicutes returned to levels similar to that of the control group. These results indicated that FMT intervention could effectively restore gut microbiota composition disrupted by DSS-induced colitis. At the genus level, the distribution varied in all studied groups (Fig. 7B). The relative abundances of the genera *Lactobacillus*, *Butyricicoccus*, *Lachnoclostridium*, *Olsenella* and *Odoribacter* reduced in DSS-induced mice but these were reversed after FMT administration. *Lactobacillus* is a well-known probiotic genus with reported beneficial effects in colitis mice [45, 48]. In fact, a previous study showed that the abundance of the genus *Butyricicoccus* decreased in IBD patients [49]. Other research findings indicate that the abundances of the genera *Olsenella* and *Odoribacter* were reduced in IBD patients [50, 51]. Observations from the present study are in agreement with these postulations, thus giving further insights into the applications of FMT. In contrast, the *Helicobacter*, *Bacteroides* and *Clostridium* genera were upregulated in the DSS group, but these were suppressed by FMT intervention. *Clostridium* is a community of gram-positive bacteria that includes several significant human pathogens [52]. *Helicobacter* is a genus of gram-negative bacteria that comprises gastrointestinal human pathogens with particular lipid A structures [53]. As reported, these two genera were enhanced in IBD [54, 55]. Supporting this, similar variations were also found in UC induced by DSS in other reports [29, 48].

FMT is a procedure in which large amounts of gut microbiota from healthy donors are transplanted to the gastrointestinal tract of dysbacteriosis recipients [56]. Our study showed that FMT administration could elevate the relative abundances of the genera *Lactobacillus, Butyricicoccus, Lachnoclostridium, Olsenella* and *Odoribacter*, but lower the genera *Helicobacter*, *Bacteroides* and *Clostridium.* These complex changes of gut microbiota contribute to the enhancement of functional bacteria via the colonization ability and create environmental conditions sufficient to inhibit the growth of pathogenic bacteria to help redress intestinal dysbacteriosis and reconstitute functional intestinal microecology [57].

### Effects of FMT on SCFA Production

**Fig. 8:**
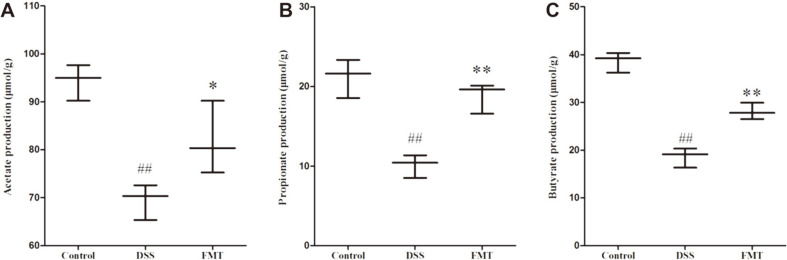
Changes of SCFAs in intestinal contents. Data are presented as mean ± SD. ##*p* < 0.01 and #*p* < 0.05 vs the control group. (**A**) acetate, (**B**) propionate, and (**C**) butyrate. **p < 0.01 and *p < 0.05 vs the DSS model group.

SCFAs act as key bacterial metabolites, mainly composed of acetate, propionate and butyrate in the intestine, which are beneficial for human health [58, 59]. As aforementioned, the relative abundances of gut microbiota related to SCFA production vary, therefore, the concentrations of SCFAs in all groups were determined using GC- MS. As shown in Fig. 8, the levels of acetate, propionate and butyrate were significantly decreased in the DSS group when compared to the control group. Compared with the DSS group, FMT intervention increased acetate, propionate and butyrate production in UC mice. These findings indicated that FMT intervention could enhance SCFA production, which is in accordance with the increased abundances of SCFA-producing microbiota. *Lactobacillus* bacteria could increase the abundances of butyrate-producing strains [60], and that lactate from *Lactobacillus* could promote butyrate production in feces [61]. *Butyricicoccus* are important butyric acid-producing bacteria in the gut microbiota, and have earlier shown colitis-alleviating properties by blocking the activation of the NF-κB transcription factor in lamina propria macrophages of UC sufferers [62, 63]. *Lachnoclostridium* could synthesize butyrate via the 4-aminobutyrate/succinate pathway [64]. It has been reported that most of *Olsenella* strains could produce acetic acid [65]. The genera *Odoribacter* also include some species that have been related to SCFA production [66].

Studies have showed that butyrate could activate the Nrf-2 signaling pathway to promote the transcription of genes encoding antioxidant enzymes to suppress oxidative stress [67, 68]. These findings can explain why FMT intervention could increase CAT, SOD and T-AOC levels. Moreover, in the intestinal mucosa, SCFAs exert beneficial effects on intestinal epithelial cells (IECs) and immune cells through induction of intracellular or extracellular processes [69]. They can increase the proliferative activity of IECs [70], with butyrate acting as an energy source to colonocytes and promoting epithelial barrier function [71, 72]. Thus, FMT intervention could improve the colon length, colon weight and ameliorate colon histopathological alterations observed in the DSS group.

### Effects of FMT on NF-κB Signaling Pathway

**Fig. 9:**
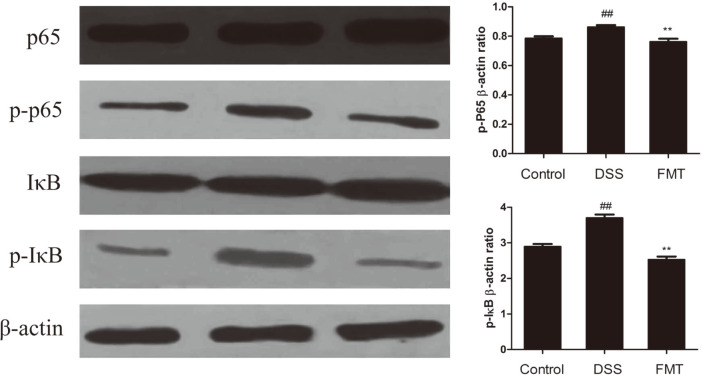
FMT modulated DSS-induced NF-κB activation in the colonic tissue. NF-κB protein levels in the colonic tissue were analyzed by western blot. β-actin was used as a control. Data are presented as mean ± SD. ##*p* < 0.01 and #*p* < 0.05 vs the control group. **p < 0.01 and *p < 0.05 vs the DSS model group.

Tedelind *et al.* suggested that acetate, propionate and butyrate could be useful in the treatment of IBD by suppressing NF-κB reporter activity [73]. Thus, the present study investigated the inhibitory mechanisms of the FMT intervention on UC through the NF-κB pathway. As shown in Fig. 9, NF-κB p65 and IκB phosphorylation levels were up-regulated significantly (*p* < 0.05) in the DSS group when compared to the control group. Interestingly, these levels were significantly (*p* < 0.05) down-regulated in the FMT group. Pro-inflammatory response occurs when IκBα is phosphorylated and p65 migrates into the nucleus [74, 75]. These results indicated that FMT intervention regulated gut microbiota and their metabolites, such as SCFAs, which could attenuate UC conditions through NF-κB signaling pathway inhibition. The NF-κB transcription factor is a key regulator that has been previously linked to genes involved in pro-inflammatory cytokine production and inflammatory cell recruitment which significantly lowers host immunity in the long run [76, 77]. Thus, FMT intervention could increase SCFA production by regulating gut microbiota, which in turn decreased pro-inflammatory cytokine production by suppressing the NF-κB signaling pathway.

In summary, the fecal microbiota transplantation (FMT) intervention showed protective effects against UC in vivo by improving the levels of DAI, relieving colon pathological damages, and reducing key cytokine expression and oxidative status in colon. Furthermore, gut microbiota functionalities were restored to normalcy after FMT intervention, leading to upregulation in SCFA levels, thus inhibiting the activation of the NF-κB signaling pathway to ameliorate UC.

## Supplemental Materials



Supplementary data for this paper are available on-line only at http://jmb.or.kr.

## Figures and Tables

**Fig. 4:**
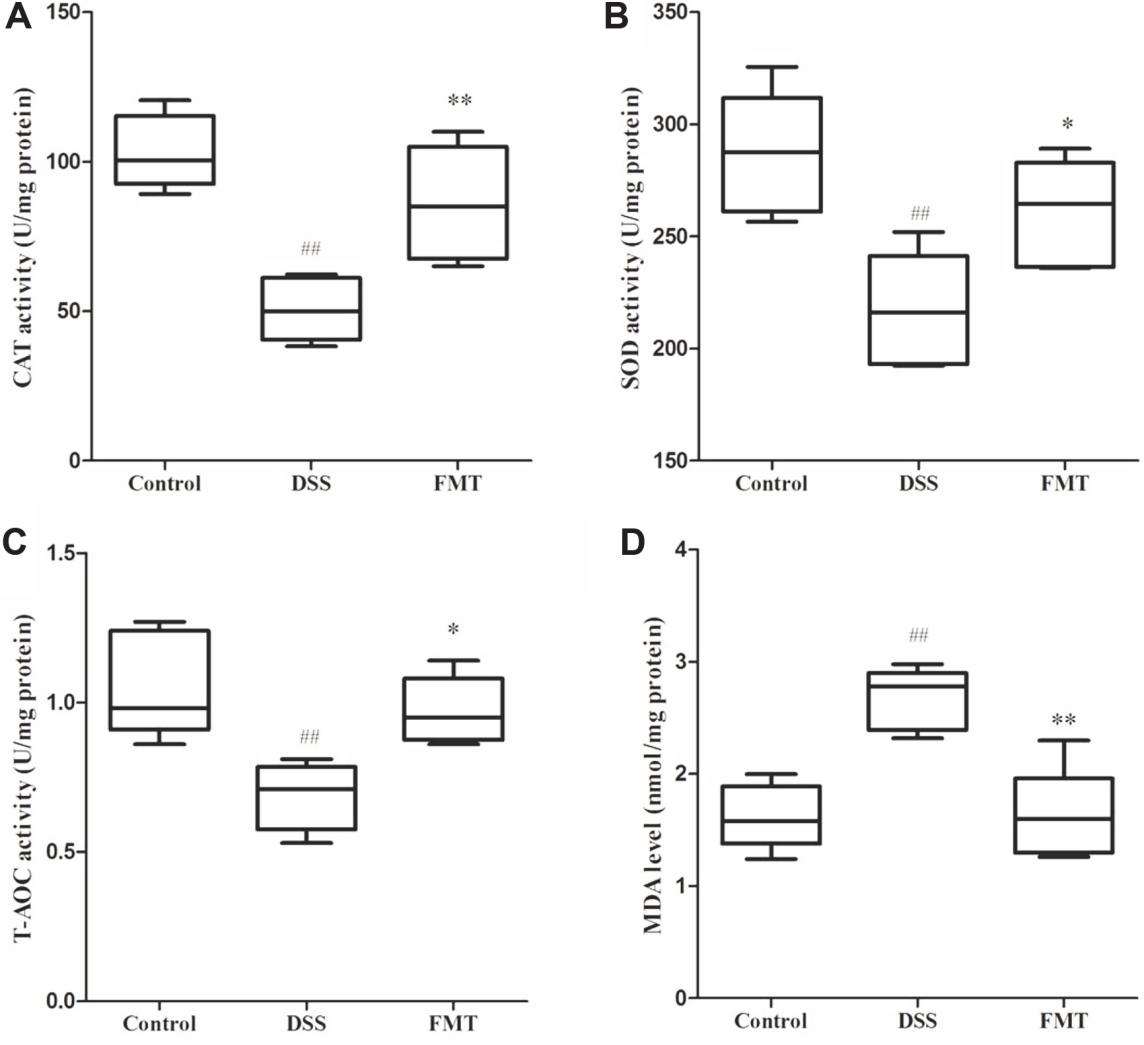
Effects of FMT intervention on colonic oxidative stress parameters in DSS-induced colitis mouse. (**A**) CAT, (**B**) SOD, (**C**) T-AOC, and (**D**) MDA. Data are presented as mean ± SD. ##*p* < 0.01 and #*p* < 0.05 vs the control group. **p < 0.01 and *p < 0.05 vs the DSS model group.
