# Supplementary material for: Fecal Microbiota Transplantation (FMT) Alleviates Experimental Colitis in Mice by Gut Microbiota Regulation
Source: J Microbiol Biotechnol. 2020 May 13;30(8):1132–41. doi: 10.4014/jmb.2002.02044 (PMC9728197; doi:10.4014/jmb.2002.02044)
Supplement: Supplementary file 1 [file JMB-30-8-1132-supple.pdf]

https://doi.org/10.4014/jmb.2002.02044

Table S1 Scoring criteria for DAI

Score	Body weight loss	Stool consistency
0	None	Normal
1	1-5%	-
2	5-10%	Loose stools
3	10-20%	-
4	＞20%	Diarrhea, gross bleeding

Table S2 Primer sequences of mRNA for RT-qPCR.

Genes	Primer sequence (5'-3')
GAPDH	F: ATGGGAAGCTTGTCATCAACG
	R: AAGACACCAGTAGACTCCACG
TNF-α	F: TGAGGTCAATCTGCCCAAGT
	R: GGGGTCAGAGTAAAGGGGTC
IL-6	F: CTGCAAGAGACTTCCATCCAG
	R: AGTGGTATAGACAGGTCTGTTGG
IL-1β	F: GGAGAAGCTGTGGACGCTA
	R: GCTGATGTACCAGTTGGGGA
